# Support Vector Machine Classifier for Estrogen Receptor Positive and Negative Early-Onset Breast Cancer

**DOI:** 10.1371/journal.pone.0068606

**Published:** 2013-07-19

**Authors:** Rosanna Upstill-Goddard, Diana Eccles, Sarah Ennis, Sajjad Rafiq, William Tapper, Joerg Fliege, Andrew Collins

**Affiliations:** 1 Human Genetics and Cancer Sciences, Faculty of Medicine, University of Southampton, Southampton, United Kingdom; 2 Centre for Operational Research, Management Science and Information Systems, University of Southampton, Southampton, United Kingdom; Health Canada and University of Ottawa, Canada

## Abstract

Two major breast cancer sub-types are defined by the expression of estrogen receptors on tumour cells. Cancers with large numbers of receptors are termed estrogen receptor positive and those with few are estrogen receptor negative. Using genome-wide single nucleotide polymorphism genotype data for a sample of early-onset breast cancer patients we developed a Support Vector Machine (SVM) classifier from 200 germline variants associated with estrogen receptor status (p<0.0005). Using a linear kernel Support Vector Machine, we achieved classification accuracy exceeding 93%. The model indicates that polygenic variation in more than 100 genes is likely to underlie the estrogen receptor phenotype in early-onset breast cancer. Functional classification of the genes involved identifies enrichment of functions linked to the immune system, which is consistent with the current understanding of the biological role of estrogen receptors in breast cancer.

## Introduction

Breast cancer sub-types may be classified according to the number of estrogen receptors present on the tumour. Tumours expressing large numbers of receptors are termed estrogen receptor positive (ER+) and, conversely, estrogen receptor negative (ER−) for few or no receptors. ER status is extremely important since ER+ cancers grow under the influence of estrogen, and may therefore respond well to hormone suppression treatments, while the proliferation of ER− cancers is not driven by estrogen and does not respond to estrogen modulation. Deroo and Korach [1] describe the “classical” (or genomic) pathway of estrogen action: an estrogen molecule binds to a receptor which induces receptor phosphorylation and dimerization to form a nuclear estrogen-ER complex [1], [2]. The transcription of target estrogen responsive genes is regulated through the binding of the estrogen-ER complex to specific estrogen response elements (EREs) located in the gene promoter region [3]. The target genes of this pathway are many and varied; the majority are crucial for normal cell physiology, growth and differentiation and can promote the growth of breast tumours under certain conditions [2], [4].

Two hypotheses seek to explain the relationship between estrogen and breast cancer. The first considers the proliferation of mammary cells stimulated by the binding of estrogen to the ER leading to an increase in the number of target cells and associated elevated risk for replication errors and acquisition of deleterious mutations during cell division and DNA replication. A second hypothesis identifies genotoxic by-products of estrogen metabolism which may lead to DNA damage and, subsequently, cancer. Evidence exists to support both hypotheses as mechanisms to initiate and promote tumour development [1]. Estrogen is necessary for breast tumour formation regardless of the receptor status of the cells and the tumour-promoting effects of estrogen are not limited to ER+ cells alone [5]. While estrogen influences the growth of ER+ tumour cells through binding receptors it is suggested that the growth of ER− tumour cells is the result of estrogen acting on cells of the tumour microenvironment which enhances angiogenesis, stromal cell recruitment and thus, tumour development and progression [5], [6].

The estrogen receptor has two forms, α and β, which are encoded by the *ESR1* and *ESR2* genes respectively. The two forms have distinct roles in breast tissue; ERα promotes cell proliferation in response to estrogen while ERβ inhibits proliferation and tumour formation [7], [8]. Single nucleotide polymorphisms (SNPs) in the *ESR1* gene have been associated with increased susceptibility to breast cancer, however they are fairly rare [9]–[11]. Variation in the *ESR2* gene may also be important in disease susceptibility however, no SNPs demonstrating a strong association with breast cancer risk have been identified [1], [12], [13]. A number of SNPs have been identified through genome wide association studies (GWAS) as being breast cancer risk SNPs. In many cases these SNPs relate to the risk of developing a particular subtype of disease, often the ER+ type [14]. Overall, the genetic basis of the estrogen receptor cancer sub-types is not well understood and worthy of further analysis [1].

We hypothesized that patients who develop ER+ and ER− tumours would show distinct constitutional genetic profiles the exploration of which could yield new insights into the biological effect of the host genomic environment on the emergence of these forms of breast cancer. We developed machine learning (ML) classifiers to explore the distinction between profiles in well characterised breast cancer cases. ML is used extensively in many scientific fields for classification purposes. ML methods have been used in genetic studies to explore the underlying genetic profile of disease and build models capable of (i) detecting gene-gene interactions; (ii) predicting disease susceptibility; (iii) predicting cancer recurrence; and (iv) predicting cancer survivability [15]. Genetic SNP data can be used to build such classification models, with high accuracy observed in many cases. Support Vector Machines (SVMs) have been shown to have excellent power and the ability to establish binary classification based on multiple features [16]. The aim of the SVM approach is to separate the data points from the two classification groups using a decision surface, called a hyperplane. The simplest classifier is a linear hyperplane but, for more complex datasets, it is necessary to map the input features into high-dimensional space using a non-linear mapping function, called a kernel function [16]. The placing of the separating hyperplane depends on maximising the margin between the hyperplane and the data points of two classes. If the input data are not cleanly separable by a hyperplane (a non-separable case, [17]), it is desirable to separate the data by the smallest sum of all classification errors: the ‘soft margin hyperplane’. In the case of genetic data linear models may be sufficient in the absence of, for example, complex underlying gene-gene interactions whilst kernel functions are most applicable otherwise. We develop here a SVM classifier which discriminates ER+ and ER− breast cancer cases which provides new insights into the biological nature of the ER+/ER− breast cancer sub-division.

## Results

### SVM classification accuracy

The overall classification accuracy of a ML classifier is a measure of how successful the method is at assigning samples to the correct class. In this study the highest classification accuracy was achieved using 200 SNPs fully genotyped in all 542 study samples (Table S1) and individually associated with the ER-negative phenotype (p<0.0005). Five kernel models were produced, all with classification accuracy exceeding 93% (Table 1). Classification accuracy was reduced when the highest ranked 50 (<86%) and 100 (<93%) SNPs were considered (Table S2). The highest classification accuracy was achieved using the radial basis function (RBF) kernel and normalized quadratic polynomial kernel: 95.95% and 95.69% respectively. In both cases 99% of the ER− cases and 89% of the ER+ cases were classified correctly. The true positive rate (number of ER+ cases correctly classified) was equal in all five models, demonstrating that they are equally successful at recognising and classifying ER+ cases in the test data. The true negative rate always exceeds 0.95, indicating that at least 95% of ER− cases are classified correctly in each model. All models are superior at classifying ER− cases compared to ER+ cases.

**Table 1:** Weka kernels and classification results using 200 SNPs with the strongest ER+/− association.

Kernel type	Percentage correctly classified	True positive rate	False positive rate	True negative rate	False negative rate	Area under ROC
Linear	93.28±3.07	0.88±0.07	0.04±0.03	0.96±0.03	0.12±0.07	0.92±0.04
Normalized quadratic polynomial	95.69±2.69	0.89±0.08	0.01±0.02	0.99±0.02	0.11±0.08	0.94±0.04
Quadratic Polynomial	93.89±3.06	0.89±0.07	0.04±0.03	0.96±0.03	0.11±0.07	0.93±0.04
Cubic Polynomial	94.54±2.94	0.89±0.07	0.03±0.03	0.97±0.03	0.11±0.07	0.93±0.04
RBF	95.95±2.61	0.89±0.07	0.01±0.02	0.99±0.02	0.11±0.07	0.94±0.04

**Figure 1:**
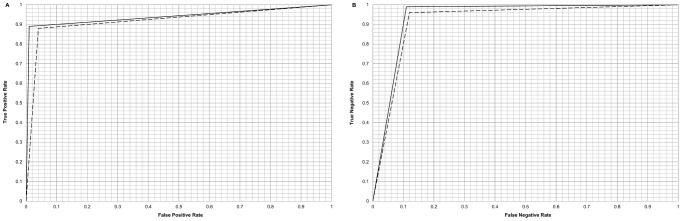
ROC curves for ER+ and ER− classification using linear and RBF kernels. ROC curves and area under ROC curve (AUC) values can be used as more robust measures of classifier accuracy beyond overall classification accuracy. (A) ROC curves for ER+ classification. (B) ROC curves for ER− classification. In both cases the linear model is represented by a dashed line and the RBF kernel model is represented by a solid line. The point on each curve corresponds to the true positive/negative and false positive/negative values obtained from 100 iterations of 10-fold cross-validation carried out on 542 samples with 200 SNP features. The ROC curve for any meaningful classifier needs to lie above the y = x line; the case where equal proportions of cases would be classified correctly and incorrectly, as would occur if class values were assigned at random.

**Figure 2:**
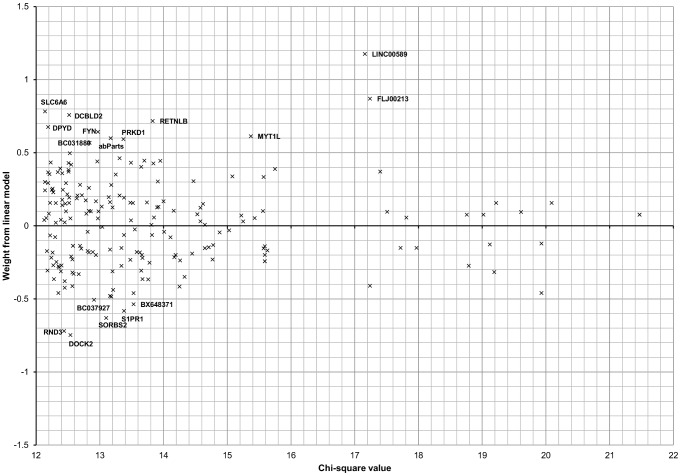
Relationship between weights under a linear classifier and chi-square values used in feature selection. SVM models were constructed on 542 study samples with genotype data for a subset of 200 SNPs chosen based on ER+/− association, determined from the chi-square statistic. SNP feature weights were obtained from the linear SVM model and used as an indicator of the importance of each feature for classification; SNPs with the largest absolute weight values are the most important for classification. Chi-square values used in feature selection and SVM classifier weight values are uncorrelated; Pearson’s correlation coefficient r = −0.026. SNPs with absolute weight values > 0.5 are annotated with the name of the gene in which they reside or are in closest proximity to.

Classifier performance was further evaluated using the receiver operating characteristic (ROC) area under curve (AUC) values which indicate these models have excellent accuracy: all exceed 0.9 (Table 1). ROC curves were produced for the linear model and RBF kernel model for both ER+ and ER− cases (Figure 1) based on true and false positive/negative values. Figure 2 shows the relationship between chi-squares for individual SNPs derived from PLINK [18], [19] and weights from the linear classification model. Variants with the largest (absolute value) weights are the most discriminating in the classifier. The input chi-squares used in feature selection (see methods) are uncorrelated with the linear SVM model weights (r = −0.026).

SVM classifiers were produced for two additional subsets of SNP features to further investigate classification accuracy. A set of 200 SNPs showing no individual association for the ER+/ER− distinction and a subset of 200 randomly selected SNPs were used to produce classification models (Table S3). Accuracy is low for both subsets (<69%) as are true positive rates in both cases (<33%). Area under ROC curve values are also very low at 0.51 or less, indicating that these models perform no better than ‘random’ which achieves an AUC of 0.5.

### DAVID functional annotation

To identify biological terms and pathways that are particularly enriched for genes represented in the classifier (Table S1) we used the DAVID annotation tool [20]–[22] DAVID identified four gene annotation clusters, three enriched pathways and 36 term annotation clusters. Of these, two gene annotation clusters and 9 term annotation clusters are particularly enriched (enrichment score ≥1.00) relative to the whole genome background. The cluster with the highest enrichment score contains genes related to the inflammatory response (Table 2) and the next highest (Table 3) shows enrichment of genes in specific pathways related to axon guidance and signalling.

**Table 2:** DAVID Annotation Clusters: Enriched gene ontology (GO) terms from the ER+/− classification.

Cluster 1: Enrichment Score: 1.97 (GO: Biological Process)				
Term	No. genes	% genes	P value	Fold Enrichment
calcium ion transport	7	6.03	0.00018	8.34
T cell proliferation	4	3.45	0.00051	25.05
di-, tri-valent inorganic cation transport	7	6.03	0.00056	6.73
T cell activation	6	5.17	0.00084	8.05
lymphocyte proliferation	4	3.45	0.00187	16.10
leukocyte proliferation	4	3.45	0.00214	15.37
mononuclear cell proliferation	4	3.45	0.00214	15.37
lymphocyte activation	6	5.17	0.00613	5.09
positive regulation of immune system process	6	5.17	0.01269	4.26
leukocyte activation	6	5.17	0.01356	4.19
cell proliferation	8	6.90	0.01360	3.10
response to abiotic stimulus	7	6.03	0.02048	3.22
cell activation	6	5.17	0.02619	3.54

Cluster 2: Enrichment Score: 1.93 (GO: Cellular Component)				
Term	No. genes	% genes	P value	Fold Enrichment
synapse	10	6.90	0.00024	4.50
cell junction	11	9.48	0.00128	3.39

**Table 3:** Significant enrichment of genes in KEGG pathway identified by DAVID.

Pathway	Genes	P value
Axon guidance	EPHA4, FYN, NRP1, NTN4, PPP3CA	0.007
T cell receptor signalling pathway	FYN, IL5, PPP3CA, PTPRC	0.027
Fc epsilon RI signalling pathway	FYN, IL5, MAP2K4	0.081

DAVID analysis was also performed for the 100 SNPs with the highest absolute classifier weights (Table S1) from the linear SVM kernel model. Similar annotation clusters were identified (data not shown) with functions relating to immune cell activation again being particularly enriched in the gene set.

## Discussion

Machine learning techniques have an important role to play in disease classification and the discovery of underlying disease mechanisms, including gene-gene interactions or signalling pathway enrichment which influences disease. Support vector machines in particular are state-of-the-art classifiers [23] with documented success at building accurate classifiers for disease versus control populations based on genetic data [24]–[26]. As discussed here, SVMs are useful for the analysis of disease sub-types given that many diseases (breast cancer included) comprise distinct sub-types with individual biology. The best resultant SVM model for ER+/ER− status in early-onset breast cancer cases successfully classifies cases into sub-types with ∼96% accuracy (Table 1), with accuracy exceeding 93% with all kernels.

Although SVM classification accuracy is an important indicator of success it can be misleading, particularly in the case of an unbalanced data set (unequal numbers of cases in the two groups), as in this study [27]. Other indicators, such as the number of cases correctly classified into each group and the area under ROC curve (AUC) values should also be considered. A ML algorithm may produce a majority-class classifier when presented with an unbalanced dataset [27]. In this situation all cases are classified as members of the majority class, making the classifier appear more accurate than in reality. For example, using this dataset, an accuracy of 68.6% can be achieved by simply classifying all 542 samples into the majority group: ER−, giving a misleading impression that the classifier is correctly identifying a reasonable proportion of the samples. However, the true positive and true negative rates, 0.00 and 1.00 respectively, identify the model as invalid. The true positive (number of true ER+ cases classified as ER+; TPR ∼89%) and true negative (number of true ER− cases classified as ER−; TNR ∼99%) results from the models in this study (Table 1) indicate that a substantial proportion of the ER+ and ER− cases are correctly classified. Therefore it is reasonable to conclude that the SVM models produced are successful as ER+/ER− classifiers and any one of the five models is suitable as a classifier for unseen data. It is evident however that the ER− cases are classified more accurately than ER+ cases, which is likely to reflect the unbalanced data (372 ER− cases versus 170 ER+ cases). The greater difficulty in classifying ER+ cases arises from the more limited variation in the SNP profile given the smaller number of cases available to the classifier.

Classifier performance can be further evaluated using receiver operator characteristic (ROC) curves which are based on the true positive and true negative rates at several different thresholds. One of the major advantages of the ROC curve is that it is unaffected by unbalanced datasets [28]. The area under ROC curve (AUC) measure [29] takes values between 0.00 and 1.00 with values closer to 1.00 indicating good performance. A random classification would produce an AUC of 0.5, the AUC values for the ER+/ER− classifier (Table 1) are in the range 0.92–0.94, suggesting excellent classification ability.

Feature selection is an important component of building a ML classifier. Much of the SNP data in these samples will not be useful for building an accurate model (Table S3) so it was necessary to select a subset of SNP features from which to build a classifier. For a review on feature selection methods available for ML algorithms see [30]. Feature selection prior to SVM implementation is essential to avoid the ‘curse of dimensionality’, which tends to arise from training of too few examples with too many variables [15]. Therefore, it is suggested that the sample-to-feature ratio should ideally exceed 5:1, which is clearly not achievable with unselected genome-wide SNP data. Machine learning theory considers the concept of VC-dimension [31]. The VC-dimension quantifies a learning machine’s *capacity* describing how complex a model can be: learning machine functions with high capacity may generate lower training error rates but require larger training sets than simpler, low capacity models. The best theoretical performance guarantee is achieved through the right balance between the accuracy attained for a given training set and the model capacity. Because analysis of genomic disease data considers potentially very large number of features (SNPs) evaluated on relatively small numbers of samples (genomes) feature selection strategies aim to reduce overfitting. Alternatives to the approach to reduce feature complexity adopted here include Recursive Feature Elimination (RFE) applied to linear SVMs using the ranked SVM weights to recursively eliminate features [32]. Such an approach has been used extensively for DNA micro-array gene expression data but has received less attention thus far for GWAS disease data.

The underlying biological nature of the genes identified as discriminators of ER+/ER− breast cancer was of particular interest in this study. To identify gene enrichment in gene groups and pathways we used the DAVID toolset. Analysis of the 139 genes that the classifier SNPs reside in, or are closest to, identified gene groups, pathways and annotation terms that were particularly enriched (Tables 2 and 3). Of the two annotation clusters with the highest enrichment scores (Table 2) it is notable that cluster 1 contains genes relating to immune/inflammatory cell activation, differentiation and proliferation. This suggests one of the distinctions between ER+ and ER− tumours relates to genetic variation in immune system pathways. The role of the immune/inflammatory response in influencing tumourigenesis and tumour progression, through the formation of an inflammatory microenvironment at the tumour site, is well characterised [33]–[36]. It has been suggested that as much as 50% of breast tumour volume comprises cells of the immune system, in particular, tumour-associated macrophages (TAMs) and tumour-infiltrating lymphocytes (TILs) [37] that establish the tumour microenvironment. Infiltrating immune cells are likely to be a major source of pro-tumourigenic factors at the tumour site because they have the capacity to release cytokines, chemokines, metalloproteases, reactive oxygen species and a number of bioactive mediators into the stroma. Furthermore, infiltrating immune cells regulate a number of processes, including enhanced cell survival, angiogenesis and suppression of anti-tumour immune responses [38] suggesting a role in both tumour development and progression. In particular, TAMs have been implicated as a source of mitogenic signals for tumour cells through cytokine secretion [39] potentially enhancing cell division and tumour growth.

The role of estrogen and estrogen receptors as regulators of proliferation and differentiation in breast tissue is well-established and is crucially important for disease progression in many cases [34], [40]. It has been suggested that infiltrating leukocytes are a major source of estrogen expression in breast tumours [41] which could contribute to disease development and progression.

The estrogen receptor status of breast cancer patients has long been recognised as a strong prognostic factor that influences patient treatment options and survival. Patients with ER− forms of the disease tend to show decreased survival rates in the first few years after diagnosis and present with more aggressive tumours [42]–[45]. However, after 10 years of disease-free survival a relapse is more likely to occur in a patient who originally presented with ER+ disease [45]. A number of other factors influence breast cancer patient survival, one of which is the infiltrating immune system cells. There is a suggested strong correlation between the infiltration of lymphocytic cells and patient survival, particularly in patients with disease onset before the age of 40 years [33]. The number of CD8^+^ T lymphocytes present at the tumour site influences patient survival, with higher numbers being associated with better survival rates. This effect is more evident in patients presenting with ER− tumours compared to ER+ tumours [46]. In contrast, TAM levels in breast tumours appear to positively correlate with aggressiveness of disease and poor prognosis [47], [48].

DAVID analysis of the gene set also identified five genes implicated in the ‘axon guidance’ pathway (Table 3). Axon guidance molecules are important in the mammary gland for maintaining normal cell proliferation and adhesion during tissue development [49] and the proximity of nerves and blood vessels in a number of tissues suggests that there may be molecular cross-talk and common cues between these structures [50]. Dysregulation of these guidance molecules in the mammary gland has been linked to breast cancer initiation and progression [49].

Genome wide association studies have identified risk-related SNPs for many diseases. Thirty-five SNPs, which lie in or near to 36 genes, are identified as breast cancer risk SNPs in the Catalog of Published Genome-Wide Association Studies [51]. From the SNPs used in the ER+/ER− classifier none of the 35 risk SNPs is present in this list nor are any of the classifier SNPs in or near the 36 catalogued genes. Thus, the SNPs identified in this study represent a set of genes not previously linked to breast cancer risk although some of the genes have been linked to roles in prognosis. Our analysis finds that variation in, or near, at least 139 genes defines the genetic background on which different estrogen receptor tumour phenotypes are most likely to arise in early onset breast cancer patients. The polygenic nature of complex phenotypes has become an emerging theme from the numerous genome-wide association studies which have identified a large number of causal variants with minor impacts on risk. A polygenic model seems appropriate to define the distinction between breast cancer sub-types such as ER+/ER−, which are likely to represent distinct forms of disease. The evidence that this distinction relates in part to genetic variation in highly complex immune system pathways reinforces the emerging concept that the presenting cancer phenotype is shaped not only by a random series of acquired somatic gene mutations but also by the stable genetic background of the individual in whom the cancer arises. Understanding interactions between the host genome and the process of oncogenesis will be an important contribution to the development of more individualised treatment and prevention approaches in the future.

## Materials and Methods

### Breast cancer samples

542 early-onset breast cancer patients were selected from the ‘**P**rospective study of **O**utcome in **S**poradic versus **H**ereditary breast cancer’ (POSH) cohort [52] of ∼3000 patients with disease onset before the age of 40 years. Germline DNA samples were genotyped for 490,732 SNPs spanning chromosomes 1 to 22. Tumours from all cases were classified for estrogen receptor status with 170 identified as ER+ and 372 as ER−. The POSH study received approval from the South and West Multi-centre Research Ethics Committee (MREC 00/6/69). Written consent was given by the patients for their information to be stored in the hospital database and used for research.

### SNP genotyping

Genotyping of the breast cancer samples was conducted using the Illumina 660-Quad SNP array. Genotyping was conducted at the Mayo Clinic, Rochester, Minnesota, USA (261 samples), and the Genome Institute of Singapore, National University of Singapore (281 samples) [53]. To ensure complete harmonisation of genotype calling, the intensity data available from both locations, in form of .idat files, were combined and used to generate genotypes using the algorithm in the genotyping module of Illumina’s Genome Studio software. A GenCall threshold of 0.15 was selected and the HumanHap660 annotation file was used. SNPs were excluded from further analysis if they had a sample minor allele frequency (MAF) below 0.01, a genotyping call rate <95% or showed significant deviation from Hardy-Weinberg equilibrium (HWE, P-value <0.0001). We used the pairwise Identity-By-State (IBS) and multidimensional scaling, implemented in PLINK v1.07 [18], [19], to confirm that patients were ethnically homogeneous. A proportion of the SNPs had missing genotypes and we used the MACH 1.0 program [54]–[56] to impute missing genotypes, where possible, based on genotype and haplotype phase data specific for CEU population available from HapMap phase 2 project. Genotype imputation was used to establish a set of SNPs with complete genotypes for testing as features in the models. However, imputation failed to resolve all genotypes for 27 SNPs with high chi-squares and these were removed from further consideration in the SVM models and replaced with the next most associated and fully genotyped SNPs in the ranked list.

### SNP feature selection

SNPs showing significant association with ER− cases were identified from the additive chi-squared association test implemented in the PLINK toolset in which ER+ samples were labelled as ‘controls’ and ER− samples were labelled as ‘cases’. Based on results from the chi-squared test all SNPs were ranked in terms of association with the ER+/− classification. Subsets of SNPs were selected as features for SVM models from the ranked list of SNPs and models were produced from subsets of 50, 100 and 200 SNPs to test utility as discriminatory factors for ER+/ER− breast cancer.

### SVM model input

The three genotypes at each SNP were converted into numeric values following [24] and [25]. Major and minor allele frequencies for each SNP were determined from all genotypes in the sample. Heterozygous genotypes were labelled 0, homozygotes for the major allele were labelled 1, and homozygotes for the minor allele labelled −1. The two classes of samples in the models were ER+ cases and ER− cases.

### Building a support vector machine classifier

Support vector machines are supervised machine learning algorithms which build models based on ‘training’ data and search for similar patterns in ‘test’ data [16]. The training set is often a subset of all samples complete with all class and feature values and the resultant model is then applied to the remaining test data. Novel data can be presented to the model and classified according to the position of the data point relative to the hyperplane constructed from the training set. The robustness and reliability of the SVM classifier can be tested using cross-validation, where the data is split into *n* equally sized sets testing *n* models. We used 10-fold cross-validation: data were divided into 10 approximately equal-sized sets and a classifier built based on the data in 9/10 of these sets. The remaining 10% of data was used as a test set to determine the accuracy of the classifier. This process was repeated 10 times with each set representing the test data once and average classification accuracy determined. We further explored 10-fold cross-validation using 100 replicates and mean accuracy from 1000 resultant models was obtained for alternative kernel models.

The SVM classification model was produced using the Weka data mining software [57], [58]. The Sequential Minimal Optimization (SMO) algorithm for training a SVM classifier was applied to the data. Five kernel models were evaluated; linear, normalized quadratic polynomial, quadratic polynomial, cubic polynomial, and radial basis function (RBF).

### Gene annotation

Annotation of sets of SNPs used in the classification models was undertaken using the ANNOVAR software [59], [60]. Gene-based annotation was carried out using the UCSC ‘Known Gene’ database. SNPs were annotated as intergenic, exonic, intronic, downstream, ncRNA intronic, ncRNA exonic, upstream, UTR3, or UTR5. For SNPs situated outside genes the closest gene was identified and gene names were taken from the HUGO Gene Nomenclature Committee database [61], [62]. A total of 139 unique gene names were linked to the set of 200 SNPs used in the final classifier (Table S1).

### Functional gene classification

Functional gene annotation clusters were identified using the ‘Gene Functional Classification’ tool in DAVID (Database for Annotation, Visualization and Integrated Discovery) [20]–[22]. DAVID determines significant enrichment of function within a submitted gene name list by contrasting with a ‘whole genome’ background. Annotation clusters were identified from the 139 genes using the ‘Functional Annotation Clustering’ tool and five annotation categories: disease, functional categories, gene ontology, pathways and protein domains. Enriched pathways were identified using only the ‘Pathways’ annotation category with BBID, BIOCARTA, and KEGG selected.

## Supporting Information

Table S1 **200 SNPs which most strongly discriminate ER+ and ER− breast cancers used in the classification models.** The weights are taken from a linear model built using one iteration of 10-fold cross-validation in the WEKA Explorer. Classification accuracy for this model was 92.4%. The magnitude of the absolute values of the SNP weights indicates importance of the SNP for classifying cases. Positive SNP weights relate to classifying ER+ cases while negative SNP weights relate to classifying ER− cases. For those SNPs that are not located within a gene the nearest gene is given and the distance of the SNP from this gene is indicated by dist = .
(DOCX)

Table S2 **Weka kernels and classification results for 100 and 50 SNPs with highest chi-squares.** Comparison of classifiers built with 100 and 50 highest ranked SNPs from PLINK chi-square test.
(DOCX)

Table S3 **Weka kernels and classification results for bottom 200 and random 200 SNPs.** Comparison of classifiers built with 200 random SNPs and the lowest ranked by PLINK chi-square test.
(DOCX)
