# Supplementary material for: Support Vector Machine Classifier for Estrogen Receptor Positive and Negative Early-Onset Breast Cancer
Source: PLoS One. 2013 Jul 19;8(7):e68606. doi: 10.1371/journal.pone.0068606 (PMC3716652; doi:10.1371/journal.pone.0068606)
Supplement: Table S1 — 200 SNPs which most strongly discriminate ER+ and ER− breast cancers used in the classification models. The weights are taken from a linear model built using one iteration of 10-fold cross-validation in the WEKA Explorer. Classification accuracy for this model was 92.4%. The magnitude of the absolute values of the SNP weights indicates importance of the SNP for classifying cases. Positive SNP weights relate to classifying ER+ cases while negative SNP weights relate to classifying ER− cases. For those SNPs that are not located within a gene the nearest gene is given and the distance of the SNP from this gene is indicated by dist = . (DOCX) [file pone.0068606.s001.docx]

**Table S1. 200 SNPs which most strongly discriminate ER+ and ER- breast cancers used in the classification models.**

| SNP rs ID | Chr. | Base-pair location | Gene or distance to nearest gene | Gene name | Gene function/description | Chi-square value | Weight from linear model |
| --- | --- | --- | --- | --- | --- | --- | --- |
| rs7713640 | 5 | 169099398 | DOCK2 | Dedicator of cytokinesis 2 | Expressed in hematopoietic cells and is involved in remodelling of the actin cytoskeleton, which is necessary for lymphocyte migration. | 12.54 | -0.7478 |
| rs10930176 | 2 | 151465458 | RND3  (dist=121278) | Rho family GTPase 3 | Binds GTP but has no GTPase activity. Possible role as a negative regulator of cytoskeletal organisation leading to loss of adhesion. | 12.44 | -0.7200 |
| rs10030246 | 4 | 186541887 | SORBS2 | Sorbin and SH3 domain containing 2 | Functions as an adapter protein that plays a role in the assembling of signaling complexes, and is a link between ABL kinases and actin cytoskeleton. | 13.10 | -0.6302 |
| rs1922987 | 1 | 101753101 | S1PR1  (dist=46025) | Sphingosine-1-phosphate receptor 1 | Receptor for sphingosine 1-phosphate ligand and suggested role in processes regulating endothelial cell differentiation. Activation of receptor induces cell-cell adhesion. | 13.38 | -0.5825 |
| rs1491477 | 8 | 126895276 | BX648371  (dist=58102) | BX648371 | Unknown | 13.53 | -0.5368 |
| rs575844 | 6 | 92395048 | BC037927 | BC037927 | Unknown | 12.91 | -0.5062 |
| rs16946160 | 13 | 92203813 | GPC5 | Glypican 5 | Cell surface heparan sulphate proteoglycan. Plays a role in cell division control and growth regulation. | 13.18 | -0.4839 |
| rs7313125 | 12 | 46464980 | SCAF11  (dist=79077) | SR-related CTD-associated factor 11 | Plays a role in pre-mRNA alternative splicing by regulating spliceosome assembly | 13.16 | -0.4802 |
| rs2034614 | 12 | 42929397 | PRICKLE1 | Prickle homolog 1 (Drosophila) | Nuclear receptor with potential role in negative regulation of Wnt signalling pathway. Implicated in nuclear trafficking of transcription repressors. | 13.53 | -0.4606 |
| rs4617179 | 8 | 4122597 | CSMD1 | CUB and Sushi multiple domains 1 | Potential suppressor of squamous cell carcinomas | 19.93 | -0.4603 |
| rs11009375 | 10 | 33706590 | NRP1  (dist=82584) | Neuropilin 1 | Involved in several signalling pathways that control cell migration. Bind many ligands and co-receptors, influencing cell survival, migration, and attraction. | 12.35 | -0.4600 |
| rs7627289 | 3 | 167348838 | WDR49 | WD repeat domain 49 | Member of the WD40-repeat family that are implicated in a wide range of processes, including signal transduction, transcription regulation, cell cycle control and apoptosis. Act as scaffolds for the assembly of protein complexes. | 13.21 | -0.4391 |
| rs3733236 | 4 | 76923998 | CXCL9 | Chemokine (C-X-C motif) ligand 9 | Potential role in T cell trafficking. | 12.45 | -0.4241 |
| rs1052651 | 12 | 96052721 | NTN4 | Netrin 4 | May play an important role in neural, kidney and vascular development. Promotes neurite elongation from olfactory bulb explants. | 14.25 | -0.4161 |
| rs1522993 | 2 | 45842064 | SRBD1  (dist=3631) | S1 RNA binding domain 1 | Unknown | 12.57 | -0.4133 |
| rs1320854 | 2 | 66363617 | AK131224  (dist=51845) | AK131224 | Unknown | 17.24 | -0.4103 |
| rs4923101 | 11 | 23520512 | ERV9-1  (dist=438843) | Endogenous retrovirus group 9, member 1 | Unknown | 12.45 | -0.3793 |
| rs10029313 | 4 | 40350147 | CHRNA9 | Cholinergic receptor, nicotinic, alpha 9 (neuronal) | Ligand-gated ionic channel family member. Forms divalent cation channels and is involved in cochlea hair cell development. | 13.76 | -0.3674 |
| rs1596623 | 12 | 46474068 | SCAF11  (dist=88165) | SR-related CTD-associated factor 11 | Plays a role in pre-mRNA alternative splicing by regulating spliceosome assembly | 12.28 | -0.3652 |
| rs6820738 | 4 | 102041236 | PPP3CA | Protein phosphatase 3, catalytic subunit, alpha isozyme | Calcium-dependent, calmodulin-stimulated protein phosphatase. This subunit may have a role in the calmodulin activation of calcineurin. | 13.67 | -0.3647 |
| rs561545 | 11 | 88101247 | CTSC  (dist=30306) | Cathepsin C | Lysosomal cysteine proteinase that activates serine proteases in immune/inflammatory cells. | 14.33 | -0.3491 |
| rs2129662 | 12 | 131084080 | RIMBP2 | RIMS binding protein 2 | Plays a role in synaptic transmission. | 12.67 | -0.3308 |
| rs10498930 | 6 | 83366352 | UBE3D  (dist=235834) | Ubiquitin protein ligase E3D | Accepts ubiquitin from specific E2 ubiquitin-conjugating enzymes, and transfers it to substrates, generally promoting their degradation by the proteasome | 12.59 | -0.3285 |
| rs1230064 | 17 | 43461460 | ARHGAP27  (dist=9809) | Rho GTPase activating protein 27 | GTPase-activating protein that inhibits Rho-like proteins. | 12.57 | -0.3197 |
| rs6082866 | 20 | 22803909 | CR618492  (dist=93277) | CR618492 | Unknown | 19.19 | -0.3169 |
| rs10505604 | 8 | 134027588 | TG | Thyroglobulin | Glycoprotein that acts as a substrate for the synthesis of thyroxine and triiodothyronine as well as storage of the inactive forms of thyroid hormone and iodine. | 12.39 | -0.3118 |
| rs6766993 | 3 | 62433496 | CADPS | Ca++-dependent secretion activator | Neural/endocrine-specific cytosolic and peripheral membrane protein required for the Ca2+-regulated exocytosis of secretory vesicles. | 13.20 | -0.3114 |
| rs7822472 | 8 | 6094406 | CR623475  (dist=166673) | CR623475 | Unknown | 13.65 | -0.3069 |
| rs6049391 | 20 | 24078128 | AK090900  (dist=102275) | AK090900 | Unknown | 12.18 | -0.3058 |
| rs4861065 | 4 | 40344395 | CHRNA9 | Cholinergic receptor, nicotinic, alpha 9 (neuronal) | Ligand-gated ionic channel family member. Forms divalent cation channels and is involved in cochlea hair cell development. | 12.36 | -0.2849 |
| rs2760415 | 9 | 5696021 | KIAA1432 | KIAA1432 | Required for phosphorylation and localization of GJA1 | 12.35 | -0.2760 |
| rs11980210 | 7 | 105448210 | ATXN7L1 | Ataxin 7-like 1 | Unknown | 13.34 | -0.2743 |
| rs4288395 | 8 | 4122628 | CSMD1 | CUB and Sushi multiple domains 1 | Potential suppressor of squamous cell carcinomas | 18.79 | -0.2730 |
| rs4782969 | 16 | 84492254 | ATP2C2 | ATPase, Ca++ transporting, type 2C, member 2 | Magnesium-dependent enzyme that catalyzes the hydrolysis of ATP coupled with the transport of calcium | 12.40 | -0.2710 |
| rs12669163 | 7 | 50278585 | IKZF1  (dist=65793) | IKAROS family zinc finger 1 (Ikaros) | Regulator of lymphocyte differentiation. | 12.27 | -0.2705 |
| rs2026604 | 1 | 153408831 | S100A7L2 | S100 calcium binding protein A7-like 2 | Unknown | 13.78 | -0.2527 |
| rs638515 | 12 | 50303927 | LOC283332 | LOC283332 | Unknown | 12.32 | -0.2480 |
| rs1515160 | 2 | 123929100 | AX747402  (dist=840522) | AX747402 | Unknown | 15.59 | -0.2431 |
| rs6706214 | 2 | 123929932 | AX747402  (dist=839690) | AX747402 | Unknown | 15.59 | -0.2431 |
| rs2052450 | 5 | 103925210 | CR610784  (dist=327983) | CR610784 | Unknown | 14.26 | -0.2369 |
| rs10906861 | 10 | 15252397 | FAM171A1  (dist=1250) | Family with sequence similarity 171, member A1 | Unknown | 13.48 | -0.2332 |
| rs1870583 | 17 | 12032944 | MAP2K4 | Mitogen-activated protein kinase kinase 4 | Dual specificity protein kinase which acts as an essential component of the MAP kinase signal transduction pathway and the stress-activated protein kinase/c-Jun N-terminal kinase (SAP/JNK) signaling pathway. | 14.77 | -0.2304 |
| rs1230073 | 17 | 43467025 | ARHGAP27  (dist=4244) | Rho GTPase activating protein 27 | GTPase-activating protein that inhibits Rho-like proteins. | 12.57 | -0.2298 |
| rs4421124 | 5 | 165002334 | BC011998  (dist=972913) | BC011998 | Unknown | 13.67 | -0.2201 |
| rs11126074 | 2 | 66352599 | AK131224  (dist=40827) | AK131224 | Unknown | 12.24 | -0.2179 |
| rs4949788 | 1 | 77750699 | AK5 | Adenylate kinase 5 | Regulates adenine nucleotide composition within cells by catalysing the transfer of phosphate groups. | 14.17 | -0.2149 |
| rs7717089 | 5 | 128643236 | ADAMTS19  (dist=152867) | ADAM metallopeptidase with thrombospondin type 1 motif, 19 | Member of the ADAMTS protein family. Suggested role in proteolysis | 12.55 | -0.2102 |
| rs11257101 | 10 | 11492868 | USP6NL  (dist=9641) | USP6 N-terminal like | Acts as a GTPase-activating protein for RAB5A. Involved in receptor trafficking. | 12.94 | -0.2002 |
| rs10850783 | 12 | 110242933 | TRPV4 | Transient receptor potential cation channel, subfamily V, member 4 | Non-selective calcium permeable cation channel probably involved in regulation of osmotic pressure. | 15.59 | -0.1996 |
| rs13261574 | 8 | 23168650 | LOXL2 | Lysyl oxidase-like 2 | Mediates the post-translational oxidative deamination of lysine residues on target proteins and is essential to the biogenesis of connective tissue. Catalyses the first step in the formation of crosslinks in extracellular matrix proteins. | 14.19 | -0.1994 |
| rs4273857 | 8 | 23173053 | LOXL2 | Lysyl oxidase-like 2 | Mediates the post-translational oxidative deamination of lysine residues on target proteins and is essential to the biogenesis of connective tissue. Catalyses the first step in the formation of crosslinks in extracellular matrix proteins. | 13.66 | -0.1994 |
| rs2454781 | 10 | 11493713 | USP6NL  (dist=8796) | USP6 N-terminal like | Acts as a GTPase-activating protein for RAB5A. Involved in receptor trafficking. | 14.45 | -0.1901 |
| rs2707237 | 2 | 38095622 | AK057187  (dist=39081) | AK057187 | Unknown | 12.26 | -0.1830 |
| rs9945952 | 18 | 54702678 | WDR7  (dist=5642) | WD repeat domain 7 | Binds Rab3A GDP/GTP exchange and activating proteins which are regulators of the control of the calcium-dependent exocytosis of neurotransmitters. | 13.63 | -0.1828 |
| rs9520707 | 13 | 108666901 | FAM155A  (dist=147441) | Family with sequence similarity 155, member A | Unknown | 12.83 | -0.1824 |
| rs211146 | 11 | 17981047 | SERGEF | Secretion regulating guanine nucleotide exchange factor | Probable guanine nucleotide exchange factor (GEF), which may be involved in the secretion process. | 13.58 | -0.1793 |
| rs211141 | 11 | 17994314 | SERGEF | Secretion regulating guanine nucleotide exchange factor | Probable guanine nucleotide exchange factor (GEF), which may be involved in the secretion process. | 13.58 | -0.1793 |
| rs2670765 | 11 | 17985273 | SERGEF | Secretion regulating guanine nucleotide exchange factor | Probable guanine nucleotide exchange factor (GEF), which may be involved in the secretion process. | 12.89 | -0.1793 |
| rs4953908 | 2 | 134960601 | MGAT5  (dist=51229) | Mannosyl (alpha-1,6-)-glycoprotein beta-1,6-N-acetyl-glucosaminyltransferase | Important for regulation of the biosynthesis of glycoprotein oligosaccharides. Catalyzes the addition of beta-1,6- N-acetylglucosamine to the alpha-linked mannose of biantennary N-linked oligosaccharides. | 12.17 | -0.1732 |
| rs366592 | 21 | 17334568 | USP25  (dist=82191) | Ubiquitin specific peptidase 25 | Deubiquitinating enzyme that processes newly synthesized Ubiquitin, recycles ubiquitin molecules or edits polyubiquitin chains and prevents proteasomal degradation of substrates. | 12.81 | -0.1726 |
| rs2431111 | 5 | 103932079 | CR610784  (dist=321114) | CR610784 | Unknown | 15.63 | -0.1689 |
| rs2148713 | 1 | 153416036 | S100A7L2  (dist=3533) | S100 calcium binding protein A7-like 2 | Unknown | 13.16 | -0.1627 |
| rs4789799 | 17 | 80533079 | FOXK2 | Forkhead box K2 | Binds purine-rich motifsin the HIV LTR and IL2 promoter. Possible role in regulating viral and cellular promoter elements. | 12.71 | -0.1565 |
| rs926745 | 20 | 22817120 | CR618492  (dist=80066) | CR618492 | Unknown | 15.57 | -0.1546 |
| rs4858909 | 3 | 162424485 | BC073807  (dist=18042) | BC073807 | Unknown | 14.65 | -0.1533 |
| rs6798611 | 3 | 162437102 | BC073807  (dist=5425) | BC073807 | Unknown | 14.65 | -0.1533 |
| rs502025 | 5 | 104022597 | CR610784  (dist=230596) | CR610784 | Unknown | 13.34 | -0.1522 |
| rs13006388 | 2 | 213825499 | IKZF2  (dist=38914) | IKAROS family zinc finger 2 (Helios) | Associates with Ikaros and functions in early hematopoietic development. | 17.97 | -0.1509 |
| rs13006331 | 2 | 213825327 | IKZF2  (dist=39086) | IKAROS family zinc finger 2 (Helios) | Associates with Ikaros and functions in early hematopoietic development. | 17.72 | -0.1509 |
| rs10515354 | 5 | 103947366 | CR610784  (dist=305827) | CR610784 | Unknown | 14.71 | -0.1469 |
| rs10515355 | 5 | 103947537 | CR610784  (dist=305656) | CR610784 | Unknown | 14.71 | -0.1469 |
| rs743562 | 5 | 131872383 | IL5  (dist=4753) | Interleukin 5 (colony-stimulating factor, eosinophil) | Induces terminal differentiation of late-developing B-cells to immunoglobulin secreting cells | 15.59 | -0.1402 |
| rs440075 | 10 | 132190059 | GLRX3  (dist=211419) | Glutaredoxin 3 | Modulates the function of protein kinase C theta and may inhibit apoptosis and play a role in cell growth. Expression of this gene may be a marker for cancer. | 12.58 | -0.1370 |
| rs396186 | 10 | 132193177 | GLRX3  (dist=214537) | Glutaredoxin 3 | Modulates the function of protein kinase C theta and may inhibit apoptosis and play a role in cell growth. Expression of this gene may be a marker for cancer. | 12.58 | -0.1370 |
| rs2075713 | 11 | 124617939 | VSIG2 | V-set and immunoglobulin domain containing 2 | Unknown | 12.69 | -0.1367 |
| rs12907348 | 15 | 49190740 | SHC4 | SHC (Src homology 2 domain containing) family, member 4 | Activates both Ras-dependent and Ras-independent migratory pathways in melanomas. | 14.78 | -0.1319 |
| rs12039894 | 1 | 74464625 | LRRIQ3  (dist=27079) | Leucine-rich repeats and IQ motif containing 3 | Unknown | 19.12 | -0.1277 |
| rs297907 | 12 | 50316818 | BC034605 | BC034605 | Unknown | 19.93 | -0.1211 |
| rs10501316 | 11 | 46090550 | PHF21A | PHD finger protein 21A | Component of the BHC complex that represses transcription of neuron-specific genes. | 14.11 | -0.0790 |
| rs2655060 | 12 | 50298535 | FAIM2 | Fas apoptotic inhibitory molecule 2 | Antiapoptotic protein which protects cells from Fas-induced apoptosis. | 12.3 | -0.0773 |
| rs10491618 | 9 | 33634591 | ANXA2  (dist=9061) | Annexin A2 | Calcium-dependent membrane-binding protein with potential role in signal transduction pathways and regulating cell growth. | 12.22 | -0.0658 |
| rs13016788 | 2 | 123930193 | AX747402  (dist=839429) | AX747402 | Unknown | 13.82 | -0.0632 |
| rs1519291 | 2 | 123954518 | AX747402  (dist=815104) | AX747402 | Unknown | 13.38 | -0.0632 |
| rs4845700 | 1 | 154981708 | ZBTB7B | Zinc finger and BTB domain containing 7B | Transcription regulator and regulator of lineage commitment of immature T-cell precursors. Transcriptional repressor of the collagen COL1A1 and COL1A2 genes. May also function as a repressor of fibronectin and other extracellular matrix genes | 14.88 | -0.0455 |
| rs1555794 | 1 | 117602077 | TTF2 | Transcription termination factor, RNA polymerase II | Has dsDNA-dependent ATPase activity and RNA polymerase II termination activity. Associates with human splicing complexes and is involved in pre-mRNA splicing. | 14.01 | -0.0415 |
| rs10919584 | 1 | 198773357 | PTPRC  (dist=46812) | Protein tyrosine phosphatase, receptor type, C | Protein tyrosine phosphatase that regulates T-cell and B-cell antigen receptor signalling. Suppresses JAK kinases, thus functioning as a cytokine receptor signalling regulator. | 12.81 | -0.0413 |
| rs1476689 | 7 | 22265585 | RAPGEF5 | Rap guanine nucleotide exchange factor (GEF) 5 | Guanine nucleotide exchange factor (GEF) for RAP1A, RAP2A and MRAS/M-Ras-GTP. | 15.03 | -0.0309 |
| rs1577635 | 6 | 62040305 | KHDRBS2  (dist=349560) | KH domain containing, RNA binding, signal transduction associated 2 | RNA-binding protein involved in the regulation of alternative splicing, mRNA splice site selection and exon inclusion. | 13.55 | -0.0248 |
| rs2125111 | 12 | 103003530 | IGF1  (dist=129152) | Insulin-like growth factor 1 (somatomedin C) | Involved in the mediation of growth and development. | 13.04 | -0.0075 |
| rs450798 | 12 | 50304949 | LOC283332 | LOC283332 | Unknown | 13.81 | 0.0064 |
| rs12897276 | 14 | 71111542 | TTC9 | Tetratricopeptide repeat domain 9 | May play a role in cancer cell invasion and metastasis. Hormonally regulated in breast cancer cells | 14.65 | 0.0073 |
| rs4884974 | 13 | 54895842 | BC044614  (dist=76482) | BC044614 | Unknown | 12.31 | 0.0213 |
| rs16906788 | 8 | 138146684 | FAM135B  (dist=995584) | Family with sequence similarity 135, member B | Unknown | 12.45 | 0.0237 |
| rs9359489 | 6 | 82120647 | FAM46A  (dist=334801) | Family with sequence similarity 46, member A | Unknown | 15.25 | 0.0298 |
| rs10737381 | 1 | 34996413 | GJB5  (dist=224308) | Gap junction protein, beta 5, 31.1kDa | Involved in intercellular communication related to epidermal differentiation and environmental sensing. | 14.58 | 0.0302 |
| rs7328941 | 13 | 108564192 | FAM155A  (dist=44732) | Family with sequence similarity 155, member A | Unknown | 13.49 | 0.0366 |
| rs2711775 | 3 | 163180280 | LOC647107  (dist=159191) | LOC647107 | Unknown | 12.13 | 0.0397 |
| rs10482869 | 21 | 16572437 | NRIP1  (dist=135311) | Nuclear receptor interacting protein 1 | Nuclear protein that interacts with the hormone-dependent domain of nuclear receptors. Modulates transcriptional activity of the estrogen receptor. | 12.39 | 0.0414 |
| rs843055 | 3 | 162975100 | LOC647107 | LOC647107 | Unknown | 12.97 | 0.0502 |
| rs843044 | 3 | 162986427 | LOC647107 | LOC647107 | Unknown | 12.54 | 0.0502 |
| rs407921 | 21 | 17316672 | USP25  (dist=64295) | Ubiquitin specific peptidase 25 | Deubiquitinating enzyme that processes newly synthesized Ubiquitin, recycles ubiquitin molecules or edits polyubiquitin chains and prevents proteasomal degradation of substrates. | 15.43 | 0.0524 |
| rs6677928 | 1 | 113771402 | AK123703  (dist=22527) | AK123703 | Unknown | 12.16 | 0.0529 |
| rs394119 | 12 | 50305007 | LOC283332 | LOC283332 | Unknown | 17.81 | 0.0561 |
| rs2824703 | 21 | 19566451 | CHODL | Chondrolectin | Encodes a type 1 membrane protein with a carbohydrate recognition domain characteristic. | 13.85 | 0.0566 |
| rs2292354 | 12 | 110368201 | GIT2 | G protein-coupled receptor kinase interacting ArfGAP 2 | GTPase-activating protein for the ADP ribosylation factor family. | 15.22 | 0.0704 |
| rs7628408 | 3 | 151377883 | AADACL2  (dist=73821) | Arylacetamide deacetylase-like 2 | Proposed role in a metabolic process | 21.47 | 0.0759 |
| rs1521590 | 3 | 151396537 | AADACL2  (dist=55167) | Arylacetamide deacetylase-like 2 | Proposed role in a metabolic process | 21.47 | 0.0759 |
| rs6048372 | 20 | 22804426 | CR618492  (dist=92760) | CR618492 | Unknown | 19.02 | 0.0759 |
| rs4679882 | 3 | 151355834 | AADACL2  (dist=95870) | Arylacetamide deacetylase-like 2 | Proposed role in a metabolic process | 18.76 | 0.0759 |
| rs1331147 | 10 | 91224329 | SLC16A12 | Solute carrier family 16, member 12 (monocarboxylic acid transporter 12) | Catalyzes the rapid transport of monocarboxylates across the plasma membrane | 14.53 | 0.0789 |
| rs17122097 | 10 | 91225140 | SLC16A12 | Solute carrier family 16, member 12 (monocarboxylic acid transporter 12) | Catalyzes the rapid transport of monocarboxylates across the plasma membrane | 14.53 | 0.0789 |
| rs10828316 | 10 | 22838389 | PIP4K2A | Phosphatidylinositol-5-phosphate 4-kinase, type II, alpha | Catalyzes the phosphorylation of phosphatidylinositol 5-phosphate to form phosphatidylinositol 4,5-bisphosphate. | 12.79 | 0.0831 |
| rs10828317 | 10 | 22839628 | PIP4K2A | Phosphatidylinositol-5-phosphate 4-kinase, type II, alpha | Catalyzes the phosphorylation of phosphatidylinositol 5-phosphate to form phosphatidylinositol 4,5-bisphosphate. | 12.20 | 0.0831 |
| rs2675221 | 15 | 76125378 | UBE2Q2  (dist=10244) | Ubiquitin-conjugating enzyme E2Q family member 2 | Catalyses the covalent attachment of ubiquitin to other proteins. | 19.61 | 0.0945 |
| rs584105 | 12 | 50306148 | BC034605 | BC034605 | Unknown | 17.51 | 0.0957 |
| rs2936610 | 11 | 126104559 | FAM118B | Family with sequence similarity 118, member B | Unknown | 12.98 | 0.0987 |
| rs11220405 | 11 | 126088824 | FAM118B | Family with sequence similarity 118, member B | Unknown | 12.86 | 0.0987 |
| rs2276312 | 11 | 126074192 | RPUSD4 | RNA pseudouridylate synthase domain containing 4 | Proposed role in pseudouridine synthesis | 12.47 | 0.0987 |
| rs254044 | 5 | 103961296 | CR610784  (dist=291897) | CR610784 | Unknown | 15.56 | 0.1005 |
| rs8049005 | 16 | 48177062 | ABCC12 | ATP-binding cassette, sub-family C (CFTR/MRP), member 12 | ABC transporter which transports molecules across extracellular and intracellular membranes. Increased expression of this gene is associated with breast cancer. | 12.83 | 0.1016 |
| rs8062641 | 16 | 48183299 | ABCC12 | ATP-binding cassette, sub-family C (CFTR/MRP), member 12 | ABC transporter which transports molecules across extracellular and intracellular membranes. Increased expression of this gene is associated with breast cancer. | 12.83 | 0.1016 |
| rs10830841 | 11 | 88147573 | BC038205  (dist=9976) | BC038205 | Unknown | 14.16 | 0.1026 |
| rs4146035 | 7 | 150152520 | GIMAP8 | GTPase, IMAP family member 8 | Exerts an anti-apoptotic effect in the immune system and is involved in infection response | 14.58 | 0.1235 |
| rs3760048 | 16 | 474180 | RAB11FIP3  (dist=1488) | RAB11 family interacting protein 3 (class II) | Acts as a regulator of the formation, targeting and fusion of intracellular transport vesicles. Interacts with and regulates Rab GTPases. | 13.20 | 0.1254 |
| rs161773 | 5 | 104086307 | CR610784  (dist=166886) | CR610784 | Unknown | 13.90 | 0.1257 |
| rs807043 | 10 | 102847237 | TLX1NB  (dist=1842) | TLX1 neighbor | Unknown | 13.92 | 0.1297 |
| rs2823130 | 21 | 16566350 | NRIP1  (dist=129224) | Nuclear receptor interacting protein 1 | Nuclear protein that interacts with the hormone-dependent domain of nuclear receptors. Modulates transcriptional activity of the estrogen receptor. | 13.03 | 0.1311 |
| rs4237446 | 10 | 15241923 | FAM171A1  (dist=11724) | Family with sequence similarity 171, member A1 | Unknown | 12.41 | 0.1406 |
| rs12050778 | 15 | 76126371 | UBE2Q2  (dist=9251) | Ubiquitin-conjugating enzyme E2Q family member 2 | Catalyses the covalent attachment of ubiquitin to other proteins. | 14.62 | 0.1495 |
| rs1487602 | 12 | 131103343 | RIMBP2 | RIMS binding protein 2 | Plays a role in synaptic transmission. | 12.46 | 0.1513 |
| rs4463750 | 10 | 14686790 | FAM107B | Family with sequence similarity 107, member B | Unknown | 12.31 | 0.1555 |
| rs970392 | 6 | 39229763 | KCNK5  (dist=32512) | Potassium channel, subfamily K, member 5 | pH-dependent, voltage insensitive, outwardly rectifying potassium channel. | 13.52 | 0.1558 |
| rs7648055 | 3 | 151414394 | AADACL2  (dist=37310) | Arylacetamide deacetylase-like 2 | Proposed role in a metabolic process | 20.09 | 0.1559 |
| rs7648113 | 3 | 151414310 | AADACL2  (dist=37394) | Arylacetamide deacetylase-like 2 | Proposed role in a metabolic process | 19.22 | 0.1559 |
| rs4359642 | 2 | 222150749 | EPHA4  (dist=132000) | EPH receptor A4 | Receptor tyrosine kinase with a potential role in the mediation of developmental events, particularly in the nervous system. | 12.52 | 0.1565 |
| rs2056246 | 11 | 18051446 | TPH1 | Tryptophan hydroxylase 1 | Catalyses the biosynthesis of serotonin. | 12.22 | 0.1568 |
| rs9889792 | 17 | 13626903 | AK123263  (dist=53246) | AK123263 | Unknown | 13.48 | 0.1585 |
| rs8032239 | 15 | 58505122 | ALDH1A2 | Aldehyde dehydrogenase 1 family, member A2 | Catalyses the synthesis of retinoic acid. | 13.74 | 0.1596 |
| rs494734 | 20 | 56908563 | RAB22A | RAB22A, member RAS oncogene family | May be involved in trafficking endosomal compartments. | 13.16 | 0.1618 |
| rs568531 | 12 | 121579673 | P2RX7 | Purinergic receptor P2X, ligand-gated ion channel, 7 | Receptor for ATP that acts as a ligand-gated ion channel. Responsible for ATP-dependent lysis of macrophages through the formation of membrane pores permeable to large molecules. | 14.00 | 0.1677 |
| rs1653583 | 12 | 121598652 | P2RX7 | Purinergic receptor P2X, ligand-gated ion channel, 7 | Receptor for ATP that acts as a ligand-gated ion channel. Responsible for ATP-dependent lysis of macrophages through the formation of membrane pores permeable to large molecules. | 12.94 | 0.1677 |
| rs7038242 | 9 | 28805269 | LINGO2  (dist=85966) | Leucine rich repeat and Ig domain containing 2 | Unknown | 12.78 | 0.1741 |
| rs7852834 | 9 | 28812865 | LINGO2  (dist=93562) | Leucine rich repeat and Ig domain containing 2 | Unknown | 12.78 | 0.1741 |
| rs6799331 | 3 | 25703640 | TOP2B | Topoisomerase (DNA) II beta 180kDa | Control of topological states of DNA during transcription. Catalyses the transient breakage and rejoining of DNA strands. Also involved in chromosome condensation, chromatid separation, and the relief of torsional stress that occurs during DNA transcription and replication. | 12.41 | 0.1776 |
| rs2689158 | 1 | 238907046 | LOC339535  (dist=257729) | LOC339535 | Unknown | 12.64 | 0.1877 |
| rs1861809 | 12 | 110245588 | TRPV4 | Transient receptor potential cation channel, subfamily V, member 4 | Non-selective calcium permeable cation channel probably involved in regulation of osmotic pressure. | 13.38 | 0.1923 |
| rs13340131 | 3 | 21794174 | ZNF385D  (dist=1358) | Zinc finger protein 385D | Unknown | 12.52 | 0.1929 |
| rs1523558 | 4 | 162002577 | CR595965  (dist=297528) | CR595965 | Unknown | 13.14 | 0.1957 |
| rs3740030 | 10 | 91222287 | SLC16A12 | Solute carrier family 16, member 12 (monocarboxylic acid transporter 12) | Catalyzes the rapid transport of monocarboxylates across the plasma membrane | 13.31 | 0.2075 |
| rs17122305 | 10 | 91237003 | SLC16A12 | Solute carrier family 16, member 12 (monocarboxylic acid transporter 12) | Catalyzes the rapid transport of monocarboxylates across the plasma membrane | 12.65 | 0.2075 |
| rs10747353 | 1 | 77751193 | AK5 | Adenylate kinase 5 | Regulates adenine nucleotide composition within cells by catalysing the transfer of phosphate groups. | 12.72 | 0.2089 |
| rs9883543 | 3 | 162447818 | BC073807 | BC073807 | Unknown | 12.49 | 0.2154 |
| rs2293786 | 3 | 25666485 | TOP2B | Topoisomerase (DNA) II beta 180kDa | Control of topological states of DNA during transcription. Catalyses the transient breakage and rejoining of DNA strands. Also involved in chromosome condensation, chromatid separation, and the relief of torsional stress that occurs during DNA transcription and replication. | 12.27 | 0.2294 |
| rs11705878 | 3 | 25683930 | TOP2B | Topoisomerase (DNA) II beta 180kDa | Control of topological states of DNA during transcription. Catalyses the transient breakage and rejoining of DNA strands. Also involved in chromosome condensation, chromatid separation, and the relief of torsional stress that occurs during DNA transcription and replication. | 12.27 | 0.2294 |
| rs861157 | 20 | 42397767 | GTSF1L  (dist=42125) | Gametocyte specific factor 1-like | Unknown | 12.14 | 0.2424 |
| rs11962201 | 6 | 53489402 | AK126334  (dist=7435) | AK126334 | Unknown | 12.25 | 0.2454 |
| rs11745512 | 5 | 164055678 | BC011998  (dist=26257) | BC011998 | Unknown | 12.42 | 0.2462 |
| rs1577608 | 1 | 18708402 | IGSF21  (dist=3426) | Immunoglobin superfamily, member 21 | May act as a receptor in immune response pathways. | 12.26 | 0.2540 |
| rs4983411 | 14 | 105916797 | MTA1 | Metastasis associated 1 | May be involved in the regulation of transcription, which may result from chromatin remodelling. | 12.83 | 0.2591 |
| rs995815 | 20 | 54530910 | CBLN4  (dist=41587) | Cerebellin 4 precursor | Involved in regulation of neurexin signalling during synapse development. | 13.18 | 0.2788 |
| rs4759493 | 12 | 131042460 | RIMBP2 | RIMS binding protein 2 | Plays a role in synaptic transmission. | 12.69 | 0.2803 |
| rs2836912 | 21 | 40506194 | PSMG1  (dist=41196) | Proteasome (prosome, macropain) assembly chaperone 1 | Chaperone protein which promotes assembly of the 20S proteasome as part of a heterodimer with PSMG2. | 12.47 | 0.2918 |
| rs6850890 | 4 | 118387036 | TRAM1L1  (dist=380300) | Translocation associated membrane protein 1-like 1 | Required for the translocation of secretory proteins across the endoplasmic reticulum membrane | 12.19 | 0.2925 |
| rs1034461 | 22 | 26331185 | MYO18B | Myosin XVIIIB | May regulate muscle-specific genes and may influence intracellular trafficking, depending on localisation. May play a role in the control of tumour development and progression. | 12.14 | 0.2997 |
| rs2442477 | 8 | 6354270 | MCPH1 | Microcephalin 1 | Encodes a DNA damage response protein that may play a role in G2/M checkpoint arrest. | 13.91 | 0.3030 |
| rs432519 | 7 | 151013263 | NUB1  (dist=25595) | Negative regulator of ubiquitin-like proteins 1 | Specific down-regulator of the NEDD8 conjugation system. Recruits NEDD8, UBD, and their conjugates to the proteasome for degradation. | 14.47 | 0.3052 |
| rs2706399 | 5 | 131867702 | IL5  (dist=9434) | Interleukin 5 (colony-stimulating factor, eosinophil) | Induces terminal differentiation of late-developing B-cells to immunoglobulin secreting cells | 15.57 | 0.3334 |
| rs9555336 | 13 | 107904621 | FAM155A | Family with sequence similarity 155, member A | Unknown | 15.08 | 0.3379 |
| rs3136146 | 16 | 14028379 | ERCC4 | Excision repair cross-complementing rodent repair deficiency, complementation group 4 | Complexes with ERCC1 to form a structure-specific DNA repair endonuclease responsible for the 5-prime incision during DNA repair. | 13.25 | 0.3509 |
| rs9356859 | 6 | 23347228 | HDGFL1  (dist=776479) | Hepatoma derived growth factor-like 1 | Unknown | 12.21 | 0.3533 |
| rs1174966 | 7 | 22046072 | CDCA7L  (dist=60530) | Cell division cycle associated 7-like | Plays a role in transcriptional regulation and gene expression. Important for oncogenic role in mediating the full transforming effect of MYC in medulloblastoma cells. Involved in apoptotic signalling pathways | 12.41 | 0.3595 |
| rs4074228 | 12 | 118987597 | SUDS3  (dist=131758) | Suppressor of defective silencing 3 homolog (S. cerevisiae) | Subunit of the histone deactylase-dependent SIN3A corepressor complex. May have a potential role in tumour suppressor pathways. | 12.19 | 0.3656 |
| rs4424536 | 1 | 59544435 | LOC729467  (dist=53175) | LOC729467 | Unknown | 12.34 | 0.3670 |
| rs4726411 | 7 | 154074097 | DPP6 | Dipeptidyl-peptidase 6 | Binds specific voltage-gated potassium channels and alters their expression and biophysical properties. | 12.51 | 0.3702 |
| rs2513421 | 11 | 88133201 | BC038205  (dist=24348) | BC038205 | Unknown | 17.40 | 0.3703 |
| rs2767326 | 1 | 117376448 | CD2  (dist=64598) | CD2 molecule | T cell surface antigen. | 12.51 | 0.3817 |
| rs16908031 | 10 | 57707708 | PCDH15  (dist=320006) | Protocadherin-related 15 | Calcium-dependent cell-adhesion protein. Essential for maintenance of normal retinal and cochlear function. | 15.75 | 0.3881 |
| rs1367002 | 11 | 20871798 | NELL1 | NEL-like 1 (chicken) | Plays a role in the control of cell growth and differentiation. | 12.38 | 0.3922 |
| rs1174965 | 7 | 22046015 | CDCA7L  (dist=60473) | Cell division cycle associated 7-like | Plays a role in transcriptional regulation and gene expression. Has an oncogenic role in mediating the transforming effect of MYC in medulloblastoma cells. Involved in apoptotic signalling pathways | 13.65 | 0.4035 |
| rs1033975 | 1 | 68567848 | AK096081/  AK124028 | AK096081/AK124028 | Unknown | 12.55 | 0.4185 |
| rs773620 | 1 | 113785301 | AK123703  (dist=36426) | AK123703 | Unknown | 13.84 | 0.4270 |
| rs7096374 | 10 | 8484113 | BC031880  (dist=173845) | BC031880 | Unknown | 12.51 | 0.4298 |
| rs10139234 | 14 | 71123560 | TTC9 | Tetratricopeptide repeat domain 9 | May play a role in cancer cell invasion and metastasis. Hormonally regulated in breast cancer cells | 13.49 | 0.4319 |
| rs6499323 | 16 | 70624483 | SF3B3  (dist=12913) | Splicing factor 3b, subunit 3, 130kDa | Subunit of the splicing factor SF3B. Involved in binding pre-mRNA upstream of the intron’s branch site. May function in chromatin modification, transcription, splicing, and DNA repair. | 12.23 | 0.4331 |
| rs10021032 | 4 | 94671148 | GRID2 | Glutamate receptor, ionotropic, delta 2 | Glutamate receptor that is one of the excitatory neurotransmitter receptors in the brain. Potential role in neuronal apoptosis. | 12.96 | 0.4401 |
| rs1001776 | 16 | 4222512 | SRL  (dist=16865) | Sarcalumenin | May be involved in the regulation of calcium transport. | 13.95 | 0.4441 |
| rs4714562 | 6 | 42036850 | TAF8 | TAF8 RNA polymerase II, TATA box binding protein (TBP)-associated factor, 43kDa | Subunit of the TFIID transcription factor complex. TFIID recognises the promoters of many genes and initiates assembly of a transcription preinitiation complex containing RNA polymerase II. | 13.70 | 0.4457 |
| rs4665867 | 2 | 26739004 | OTOF | Otoferlin | May be involved in vesicle membrane fusion. Mutations in this gene cause neurosensory nonsyndromic recessive deafness. | 13.31 | 0.4619 |
| rs4807753 | 19 | 5399090 | ZNRF4  (dist=56336) | Zinc and ring finger 4 | Unknown | 12.53 | 0.4961 |
| rs10905371 | 10 | 8480044 | BC031880  (dist=169776) | BC031880 | Unknown | 12.84 | 0.5678 |
| rs17096099 | 14 | 30332962 | PRKD1 | Protein kinase D1 | Serine/threonine-protein kinase that regulates membrane receptor signalling, Golgi transport, protection from oxidative stress at the mitochondria, gene transcription, and regulation of cell shape, motility, and adhesion. | 13.37 | 0.5931 |
| rs10854759 | 22 | 22889528 | abParts | abParts | Unknown | 13.17 | 0.5990 |
| rs7600426 | 2 | 2263952 | MYT1L | Myelin transcription factor 1-like | May function as a panneural transcription factor associated with neuronal differentiation. May play a role in the development of neurons and oligodendrogalia in the CNS | 15.37 | 0.6126 |
| rs9384805 | 6 | 112101725 | FYN | FYN oncogene related to SRC, FGR, YES | Membrane-associated tyrosine kinase involved in cell growth control. Member of the protein-tyrosine kinase oncogene family. | 12.97 | 0.6422 |
| rs290826 | 1 | 97473707 | DPYD  (dist=69595) | Dihydropyrimidine dehydrogenase | Pyrimidine catabolic enzyme involved in catabolism of uracil and thymidine. | 12.19 | 0.6759 |
| rs4146282 | 3 | 108497048 | RETNLB  (dist=20918) | Resistin like beta | Probable hormone. | 13.83 | 0.7170 |
| rs3796133 | 3 | 98517843 | DCBLD2 | Discoidin, CUB and LCCL domain containing 2 | Proposed role in cell adhesion, wound healing, intracellular receptor mediated signalling pathway, and negative regulation of cell growth | 12.52 | 0.7584 |
| rs3773162 | 3 | 14526031 | SLC6A6 | Solute carrier family 6 (neurotransmitter transporter, taurine), member 6 | Required for the uptake of taurine. | 12.14 | 0.7827 |
| rs4936947 | 11 | 124498785 | FLJ00213 | FLJ00213 | Unknown | 17.24 | 0.8694 |
| rs4732990 | 8 | 29536211 | LINC00589  (dist=42567) | Long intergenic non-protein coding RNA 589 | Unknown | 17.16 | 1.1758 |
