# Supplementary material for: Support Vector Machine Classifier for Estrogen Receptor Positive and Negative Early-Onset Breast Cancer
Source: PLoS One. 2013 Jul 19;8(7):e68606. doi: 10.1371/journal.pone.0068606 (PMC3716652; doi:10.1371/journal.pone.0068606)
Supplement: Table S2 — Weka kernels and classification results for 100 and 50 SNPs with highest chi-squares. Comparison of classifiers built with 100 and 50 highest ranked SNPs from PLINK chi-square test. (DOCX) [file pone.0068606.s002.docx]

**Table S2. Weka kernels and classification results for 100 and 50 SNPs with highest chi-squares.**

| **Top 100 SNPs** | | | | | | |
| --- | --- | --- | --- | --- | --- | --- |
| Kernel type | Percentage correctly classified | True positive rate | False positive rate | True negative rate | False negative rate | Area under ROC |
| Linear | 90.00 ± 3.93 | 0.83 ± 0.09 | 0.07 ± 0.04 | 0.93 ± 0.04 | 0.17 ± 0.09 | 0.88 ± 0.05 |
| Normalized quadratic polynomial | 92.42 ± 3.25 | 0.82 ± 0.09 | 0.03 ± 0.03 | 0.97 ± 0.03 | 0.18 ± 0.09 | 0.90 ± 0.05 |
| Quadratic polynomial | 88.30 ± 4.15 | 0.81 ± 0.10 | 0.08 ± 0.05 | 0.92 ± 0.05 | 0.19 ± 0.10 | 0.86 ± 0.05 |
| Cubic polynomial | 89.42 ± 4.08 | 0.82 ± 0.09 | 0.07 ± 0.04 | 0.93 ± 0.04 | 0.18 ± 0.09 | 0.87 ± 0.05 |
| RBF | 91.25 ± 3.36 | 0.77 ± 0.10 | 0.02 ± 0.02 | 0.98 ± 0.02 | 0.23 ± 0.10 | 0.87 ± 0.05 |
| **Top 50 SNPs** | | | | | | |
| Kernel type | Percentage correctly classified | True positive rate | False positive rate | True negative rate | False negative rate | Area under ROC |
| Linear | 85.22 ± 4.57 | 0.73 ± 0.11 | 0.09 ± 0.04 | 0.91 ± 0.04 | 0.27 ± 0.11 | 0.82 ± 0.06 |
| Normalized quadratic polynomial | 85.09 ± 4.31 | 0.64 ± 0.11 | 0.05 ± 0.04 | 0.95 ± 0.04 | 0.36 ± 0.11 | 0.79 ± 0.06 |
| Quadratic polynomial | 78.15 ± 5.07 | 0.66 ± 0.11 | 0.16 ± 0.06 | 0.84 ± 0.06 | 0.34 ± 0.11 | 0.75 ± 0.06 |
| Cubic polynomial | 78.40 ± 5.05 | 0.66 ± 0.11 | 0.16 ± 0.06 | 0.84 ± 0.06 | 0.34 ± 0.11 | 0.75 ± 0.06 |
| RBF | 79.81 ± 3.38 | 0.38 ± 0.11 | 0.01 ± 0.02 | 0.99 ± 0.02 | 0.62 ± 0.11 | 0.69 ± 0.05 |
