# Supplementary material for: Support Vector Machine Classifier for Estrogen Receptor Positive and Negative Early-Onset Breast Cancer
Source: PLoS One. 2013 Jul 19;8(7):e68606. doi: 10.1371/journal.pone.0068606 (PMC3716652; doi:10.1371/journal.pone.0068606)
Supplement: Table S3 — Weka kernels and classification results for bottom 200 and random 200 SNPs. Comparison of classifiers built with 200 random SNPs and the lowest ranked by PLINK chi-square test. (DOCX) [file pone.0068606.s003.docx]

**Table S3. Weka kernels and classification results for bottom 200 and random 200 SNPs.**

| **Bottom 200 SNPs** | | | | | | |
| --- | --- | --- | --- | --- | --- | --- |
| Kernel type | Percentage correctly classified | True positive rate | False positive rate | True negative rate | False negative rate | Area under ROC |
| Linear | 66.75 ± 4.61 | 0.00 ± 0.00 | 0.03 ± 0.07 | 0.97 ± 0.07 | 1.00 ± 0.00 | 0.49 ± 0.03 |
| Normalized quadratic polynomial | 68.63 ± 0.23 | 0.00 ± 0.00 | 0.00 ± 0.00 | 1.00 ± 0.00 | 1.00 ± 0.00 | 0.50 ± 0.00 |
| Quadratic polynomial | 37.37 ± 5.98 | 0.00 ± 0.01 | 0.46 ± 0.09 | 0.54 ± 0.09 | 1.00 ± 0.01 | 0.27 ± 0.04 |
| Cubic polynomial | 40.46 ± 5.93 | 0.00 ± 0.01 | 0.41 ± 0.09 | 0.59 ± 0.09 | 1.00 ± 0.01 | 0.30 ± 0.04 |
| RBF | 68.63 ± 0.23 | 0.00 ± 0.00 | 0.00 ± 0.00 | 1.00 ± 0.00 | 1.00 ± 0.00 | 0.50 ± 0.00 |
| **Random 200 SNPs** | | | | | | |
| Kernel type | Percentage correctly classified | True positive rate | False positive rate | True negative rate | False negative rate | Area under ROC |
| Linear | 58.17 ± 6.03 | 0.32 ± 0.11 | 0.30 ± 0.08 | 0.70 ± 0.08 | 0.68 ± 0.11 | 0.51 ± 0.06 |
| Normalized quadratic polynomial | 68.63 ± 0.23 | 0.00 ± 0.00 | 0.00 ± 0.00 | 1.00 ± 0.00 | 1.00 ± 0.00 | 0.50 ± 0.00 |
| Quadratic polynomial | 57.47 ± 6.02 | 0.33 ± 0.11 | 0.31 ± 0.08 | 0.69 ± 0.08 | 0.67 ± 0.11 | 0.51 ± 0.06 |
| Cubic polynomial | 58.42 ± 5.85 | 0.32 ± 0.11 | 0.29 ± 0.08 | 0.71 ± 0.08 | 0.69 ± 0.11 | 0.51 ± 0.06 |
| RBF | 68.63 ± 0.23 | 0.00 ± 0.00 | 0.00 ± 0.00 | 1.00 ± 0.00 | 1.00 ± 0.00 | 0.50 ± 0.00 |
